# Impact of Vasectomy on the Development and Progression of Prostate Cancer: Preclinical Evidence

**DOI:** 10.3390/cancers12082295

**Published:** 2020-08-15

**Authors:** Takashi Kawahara, Yuki Teramoto, Yi Li, Hitoshi Ishiguro, Jennifer Gordetsky, Zhiming Yang, Hiroshi Miyamoto

**Affiliations:** 1 Department of Pathology & Laboratory Medicine, University of Rochester Medical Center, Rochester, NY 14642, USA; takashi_tk2001@yahoo.co.jp (T.K.); yuki_teramoto@urmc.rochester.edu (Y.T.); 13957160303@163.com (Y.L.); hitoshi.ishiguro@gmail.com (H.I.); Jennifer.b.gordetsky@vumc.org (J.G.); yangzm2000@hotmail.com (Z.Y.); 2 Department of Pathology, Johns Hopkins University School of Medicine, Baltimore, MD 21287, USA; 3 James Buchanan Brady Urological Institute, Johns Hopkins University School of Medicine, Baltimore, MD 21287, USA; 4 Departments of Urology and Renal Transplantation, Yokohama City University Medical Center, Yokohama 232-0024, Japan; 5 James P. Wilmot Cancer Institute, University of Rochester Medical Center, Rochester, NY 14642, USA; 6 Kanagawa Institute of Industrial Science and Technology, Kawasaki 210-0821, Japan; 7 Department of Urology, Yokohama City University Graduate School of Medicine, Yokohama 236-0004, Japan; 8 Department of Urology, University of Rochester Medical Center, Rochester, NY 14642, USA; 9 Departments of Pathology and Urology, Vanderbilt University Medical Center, Nashville, TN 37212, USA

**Keywords:** immunohistochemistry, prostate cancer, TRAMP model, vasectomy, ZKSCAN3

## Abstract

Some observational studies have implied a link between vasectomy and an elevated risk of prostate cancer. We investigated the impact of vasectomy on prostate cancer outgrowth, mainly using preclinical models. Neoplastic changes in the prostate were compared in transgenic TRAMP mice that underwent vasectomy vs. sham surgery performed at 4 weeks of age. One of the molecules identified by DNA microarray (i.e., ZKSCAN3) was then assessed in radical prostatectomy specimens and human prostate cancer lines. At 24 weeks, gross tumor (*p* = 0.089) and poorly differentiated adenocarcinoma (*p* = 0.036) occurred more often in vasectomized mice. Vasectomy significantly induced *ZKSCAN3* expression in prostate tissues from C57BL/6 mice and prostate cancers from TRAMP mice. Immunohistochemistry showed increased ZKSCAN3 expression in adenocarcinoma vs. prostatic intraepithelial neoplasia (PIN), PIN vs. non-neoplastic prostate, Grade Group ≥3 vs. ≤2 tumors, pT3 vs. pT2 tumors, pN1 vs. pN0 tumors, and prostate cancer from patients with a history of vasectomy. Additionally, strong (2+/3+) ZKSCAN3 expression (*p* = 0.002), as an independent prognosticator, or vasectomy (*p* = 0.072) was associated with the risk of tumor recurrence. In prostate cancer lines, ZKSCAN3 silencing resulted in significant decreases in cell proliferation/migration/invasion. These findings suggest that there might be an association between vasectomy and the development and progression of prostate cancer, with up-regulation of ZKSCAN3 expression as a potential underlying mechanism.

## 1. Introduction

Vasectomy is a simple surgical procedure used for male sterilization. From the National Survey of Family Growth in the United States, at least 500,000 American men were estimated to annually undergo vasectomy as their permanent form of contraception [1,2]. Historically speaking, vasectomy has also been performed at the time of prostatic surgery, such as transurethral resection for benign prostatic hyperplasia, to provide protection against postoperative acute epididymitis [3].

There are numerous studies assessing the potential health risks associated with vasectomy, including subsequent development of prostate cancer. Indeed, a substantial number of cohort and case-control studies have shown an elevated risk of prostate cancer in men who have undergone vasectomy, with an odds ratio (OR) up to 5.3 in US studies (Figure S1) [4,5,6,7,8,9,10,11,12,13,14,15,16,17,18,19,20,21,22,23,24,25]. However, some of these studies have been criticized as suffering from detection bias or surveillance bias. In a few studies, considerable increases in the incidence of high-grade and/or lethal prostate cancer in vasectomized men have also been documented [19,26]. One such study demonstrated an OR of 1.22 for Gleason score ≥8 cancer (*p* = 0.02) and an OR of 1.19 for lethal disease (*p* = 0.05) in men who had undergone vasectomy [19]. The findings in these observational studies have thus suggested that vasectomy promotes prostate carcinogenesis, particularly the development of aggressive tumor. By contrast, two smaller studies suggested a link between vasectomy and lower grade prostate cancer [15,16], possibly related to selection bias (i.e., vasectomy recipients typically had good access to health care/urologists and were more likely to be screened for prostate cancer). Accordingly, the association between vasectomy and prostate cancer is of concern and needs further assessments, especially in preclinical models.

We have previously demonstrated that semenogelin-I, a major structural protein in human semen, induces prostate cancer progression via functioning as an androgen receptor co-activator [27,28,29,30]. It is therefore entirely possible that vasectomy alters the expression or secretion pattern of seminal plasma proteins and/or other molecules, and thereby promotes (or may inhibit) prostate cancer outgrowth. The present study aimed to investigate whether vasectomy affects the development and progression of prostate cancer as well as underlying mechanisms, using animal and cell line models in addition to surgical specimens.

## 2. Results

### 2.1. Vasectomy in TRAMP Mice

**Figure 1:**
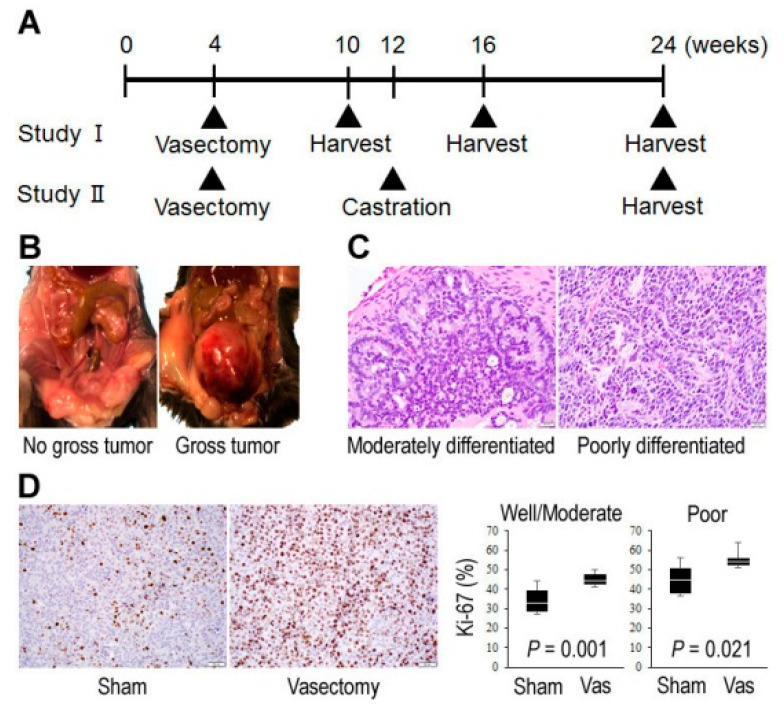
Effects of vasectomy on tumor outgrowth in TRAMP mice. (**A**) Design of Studies I and II. Vasectomy or sham surgery was performed at the age of 4 weeks. In groups of mice (Study II), bilateral orchiectomy or sham surgery was additionally performed at the age of 12 weeks. Macroscopic assessment was then performed at 10/16/24 (Study I) or 24 (Study II) weeks, and at the same time tissues were harvested for microscopic analysis. (**B**) Representative gross findings. (**C**) Representative histological findings (original magnification: ×400). (**D**) Immunohistochemistry of Ki-67 in prostate cancer (original magnification: ×200). The proliferation index (%) was determined by counting at least 500 Ki-67-positive/negative cancer cells in each mouse (*n* = 2 for sham/poorly differentiated at 24 weeks; *n* = 3 for other groups at 24 weeks).

We first compared histopathological findings at different time points within the prostate from TRAMP (Transgenic adenocarcinoma of mouse prostate) mice (undergoing vasectomy vs. sham surgery; see Figure 1A) where neoplastic changes are sequentially developed [31].

Table 1 summarizes the incidence of macroscopic and microscopic tumors at 10, 16, and 24 weeks in Study I. At 24 weeks, gross tumor (see Figure 1B) was seen more often in vasectomized mice (58%) compared with controls (17%), but the difference was not statistically significant (*p* = 0.089). Histologically, prostatic intraepithelial neoplasia (PIN) lesions were seen in all the mice in both groups even at 10 weeks, while adenocarcinoma was eventually found in all of them by 24 weeks. At 24 weeks, control mice more often developed well/moderately differentiated carcinomas (*p* = 0.036), while vasectomized mice more often developed poorly differentiated carcinomas (*p* = 0.036) (see Figure 1C). There was no significant difference (*p* = 0.640) in the incidence of metastasis (lymph node: *n* = 5; kidney: *n* = 1) between control (17%) and vasectomized (33%) mice. At earlier time points, no significant differences in the development of gross tumor, well/moderately differentiated carcinoma, or poorly differentiated carcinoma were observed between the two groups. We further compared cell proliferation indices in tumors via Ki-67 immunohistochemistry. In both well/moderately carcinomas (*p* = 0.001) and poorly differentiated carcinomas (*p* = 0.021), the Ki-67 proliferation index was significantly higher in vasectomized mice than in controls (Figure 1D).

In Study II, while castration at 12 weeks of age was anticipated to rapidly promote aggressive castration-resistant tumor (except a subset (e.g., 20%) with “cure” [31]), only 1 (4%) developed gross tumor and 3 (12%) developed poorly differentiated carcinoma at 24 weeks (Table S1). No metastatic disease was identified in any of these animals. Thus, there were no significant differences in the incidence of gross tumor, well/moderately differentiated carcinoma, poorly differentiated carcinoma, or metastasis between the sham surgery and vasectomy groups.

### 2.2. Identification of ZKSCAN3 as a Molecule whose Expression is Significantly Up-Regulated by Vasectomy

**Figure 2:**
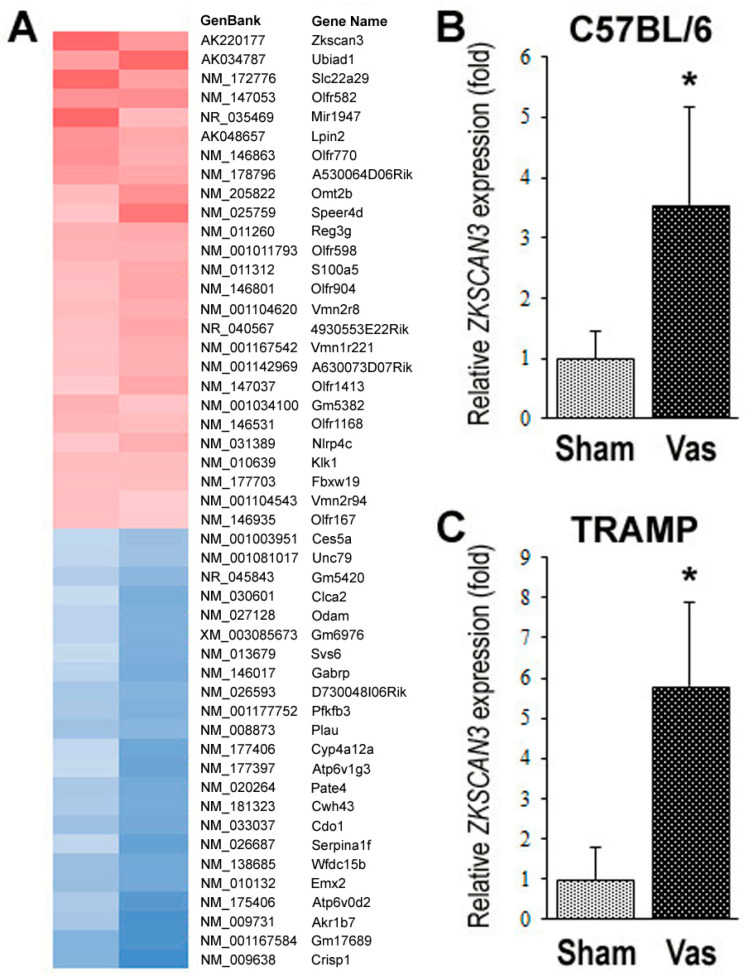
Identification of ZKSCAN3 as the one whose expression is significantly altered by vasectomy. (**A**) Gene expression was systematically compared by DNA microarray in prostate tissues from two pairs of 24-week-old male C57BL/6 mice undergoing sham surgery vs. vasectomy at 10 weeks of age. Red/blue = up/down-regulated genes, respectively, in vasectomized mice. Actual microarray data have been deposited in the NCBI Gene Expression Omnibus (GEO; http://www.ncbi.nlm.nih.gov/geo/) and are accessible through GEO Series Accession number GSE54003. Quantitative real-time RT-PCR for *ZKSCAN3*, using prostate tissues from 5 pairs of 24-week-old male C57BL/6 mice undergoing sham surgery vs. vasectomy at 10 weeks (**B**) or 8 pairs of 24-week-old male TRAMP mice undergoing sham surgery vs. vasectomy at 4 weeks (**C**). The expression of *ZKSCAN3* gene was normalized to that of *GAPDH*, and transcription amount representing the mean (+SD) is presented relative to that in the sham/control group. * *p* < 0.01 (vs. control).

We employed DNA microarray analysis in the prostate tissues from two pairs of 24-week-old C57BL/6 mice undergoing sham surgery versus vasectomy at 10 weeks. The dorsolateral lobe was used because of its correspondence to the human peripheral zone [32] where the majority of prostatic adenocarcinomas originate. Of those with absolute high signals, 26 and 23 genes were found to be considerably up- and down-regulated, respectively, by vasectomy (Figure 2A). A quantitative PCR confirmed the increase/decrease in the expression of several candidate genes in mouse prostate tissues. Of these, the expression of *ZKSCAN3* was indeed significantly up-regulated in non-neoplastic prostate tissues from 24-week-old C57BL/6 mice undergoing vasectomy at 10 weeks (Figure 2B) and in prostate cancers from 24-week-old TRAMP mice undergoing vasectomy at 4 weeks (Figure 2C). We thus decided to further investigate the function of ZKSCAN3 in prostate cancer, using surgical specimens as well as human cell lines.

### 2.3. Expression of ZKSCAN3 in Prostate Cancer Specimens

**Figure 3:**
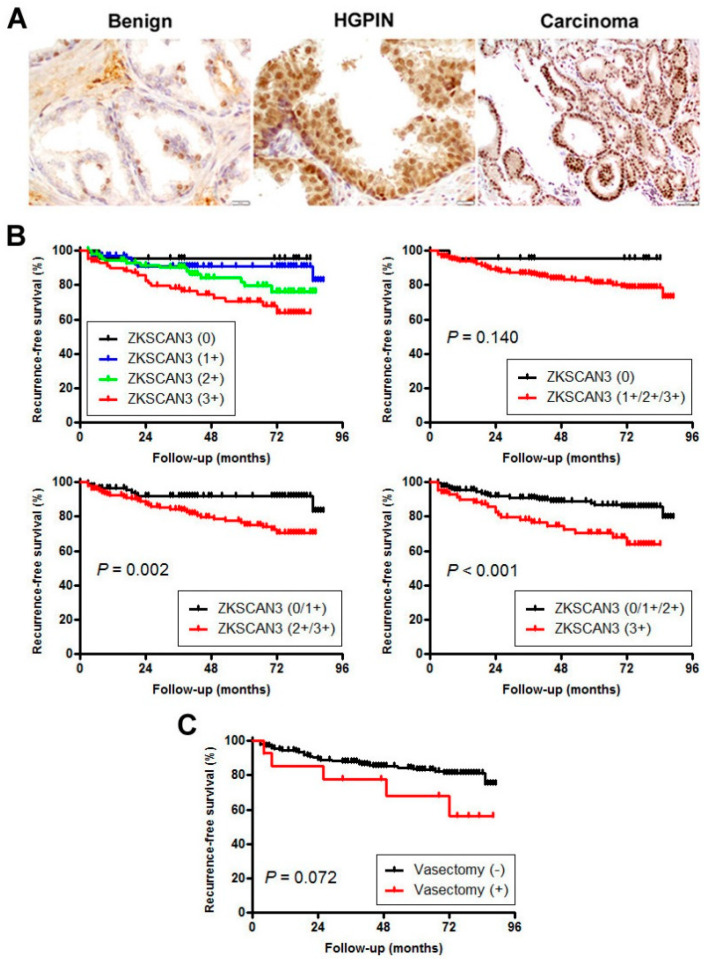
Expression of ZKSCAN3 in radical prostatectomy specimens. (**A**) Representative images of immunohistochemistry in benign prostatic tissue (original magnification: ×400), HGPIN (original magnification: ×400), and prostatic adenocarcinoma (original magnification: ×200). Kaplan-Meier analyses for recurrence-free survival, according to the levels (0: *n* = 21; 1+: *n* = 99; 2+: *n* = 95; 3+: *n* = 85) of ZKSCAN3 expression in carcinoma (**B**) or a known history of vasectomy (*n* = 14) (**C**). Biochemical recurrence after radical prostatectomy was defined as a single prostate-specific antigen (PSA) level of ≥0.2 ng/mL.

We stained immunohistochemically for ZKSCAN3 in a set of prostate tissue microarray (TMA) consisting of radical prostatectomy specimens. Positive signals were detected predominantly in the nucleus of non-neoplastic/neoplastic epithelial cells (Figure 3A). Overall, ZKSCAN3 was immunoreactive in 51 (34%) of 150 benign, 92 (63%) of 146 high-grade PIN (HGPIN), and 279 (93%) of 300 carcinoma tissues (Table 2). Thus, the positive rates or levels of ZKSCAN3 expression were significantly higher in carcinoma than in benign prostate tissue or HGPIN and in HGPIN than in benign tissue. ZKSCAN3 expression was also considerably elevated in higher grade tumors (e.g., Grade Groups 1-2 vs. 3-5, 1-3 vs. 4-5), higher pT stage tumors (e.g., pT2 vs. pT3, pT2/pT3a vs. pT3b), and lymph node-positive cases.

We then performed Kaplan-Meier analysis coupled with the log-rank test to assess possible associations of ZKSCAN3 expression with patient outcomes. Patients with ZKSCAN3(2+/3+) (*p* = 0.002) or ZKSCAN3(3+) (*p* < 0.001) tumor had a significantly higher risk of biochemical recurrence after radical prostatectomy, compared to those with ZKSCAN3(0/1+) or ZKSCAN3(0/1+/2+) tumor, respectively (Figure 3B). To see whether ZKSCAN3 expression was an independent prognostic factor, multivariate analysis was performed with Cox model, including dichotomized PSA level, Grade Group, pT stage, and pN stage. In these subgroups, ZKSCAN3 levels (0/1+ vs. 2+/3+: hazard ratio (HR) = 2.569, 95% confidence interval (CI) = 1.241–5.320, *p* = 0.011; 0/1+/2+ vs. 3+: HR = 1.766, 95% CI = 0.937–3.327, *p* = 0.079) were associated with the risk of tumor recurrence.

In the cohort included in the TMA, 14 men were found to have a history of vasectomy. The level of ZKSCAN3 expression in vasectomized patients was significantly higher than that in those without a known history of vasectomy (Table 2). The level of preoperative PSA was significantly higher in vasectomized patients, while there were no significant differences in age, Grade Group, or pT/pN stage between the two cohorts (Table S2). In addition, an association between prior vasectomy in radical prostatectomy patients and the risk of biochemical recurrence was not statistically significant (*p* = 0.072; Figure 3C).

### 2.4. ZKSCAN3 Expression in Prostate Cancer Lines and Its Silencing Effect on Cell Growth

**Figure 4:**
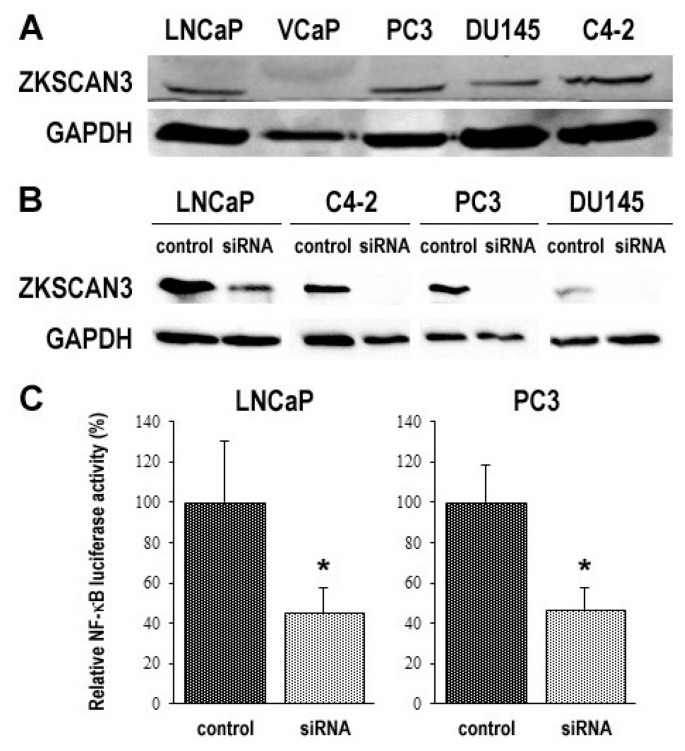
Silencing of ZKSCAN3 in prostate cancer cell lines. Western blotting of ZKSCAN3 (60 kDa) in 5 parental prostate cancer cell lines (**A**) or 4 prostate cancer cell lines transfected with either control-siRNA or ZKSCAN3-siRNA (**B**). GAPDH (37 kDa) served as an internal control. (**C**) NF-κB luciferase reporter activity in 2 prostate cancer cell lines transfected with either control-siRNA or ZKSCAN3-siRNA. The activity is presented relative to that of each control line. * *p* < 0.001 (vs. control-siRNA).

To determine the impact of ZKSCAN3 on tumor progression, we first examined its expression in 5 human prostate cancer lines. Western blot detected ZKSCAN3 signals in all of the cell lines, except VCaP (Figure 4A and Figure S2A). In the 4 ZKSCAN3-positive cell lines, we silenced ZKSCAN3 by transfection of its small interfering RNA (siRNA) (Figure 4B and Figure S2B). A luciferase assay with a reporter plasmid further showed that the transcriptional activity of NF-κB, a known downstream target of ZKSCAN3 signals [33], was significantly diminished in ZKSCAN3-siRNA cells, compared with control-siRNA cells (Figure 4C).

**Figure 5:**
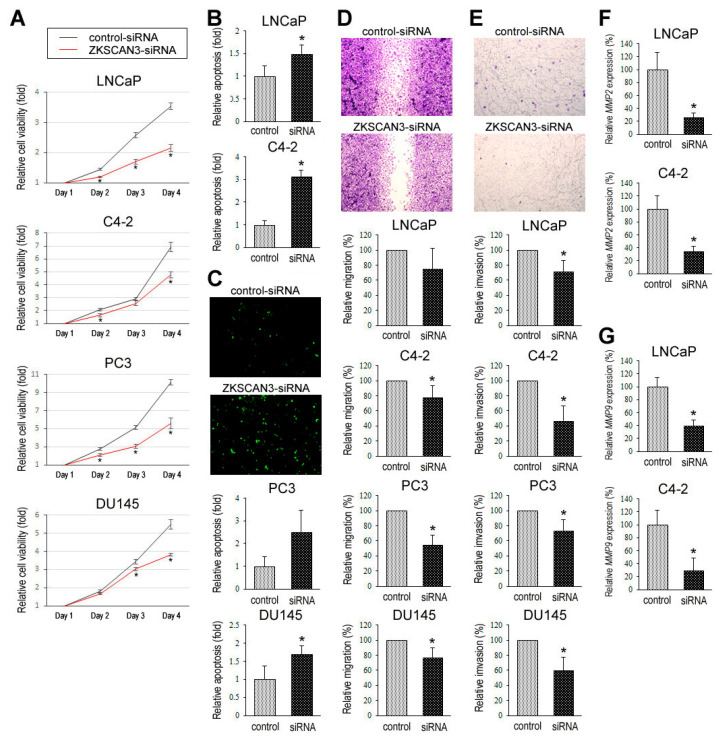
Effects of ZKSCAN3 silencing on prostate cancer cell growth. (**A**) MTT assay in 4 prostate cancer cell lines transfected with either control-siRNA or ZKSCAN3-siRNA and cultured for 1–4 days. Cell viability is presented relative to that of each control line at day 1. Each value represents the mean (+SD) of 5 determinants. * *p* < 0.05 (vs. control-siRNA). Flow cytometry (**B**) and TUNEL assay (**C**) in 2 prostate cancer cell lines transfected with either control-siRNA or ZKSCAN3-siRNA. Apoptosis is presented relative to that of each control line. Each value represents the mean (+SD) of 3 determinants. * *p* < 0.05 (vs. control-siRNA). (**D**) Wound-healing assay in 4 prostate cancer cell lines transfected with either control-siRNA or ZKSCAN3-siRNA. The cells grown to confluence were gently scratched and the wound area was measured after 24-h culture. The migration determined by the rate of cells filling the wound area (24 h/0 h) is presented relative to that of each control line. Each value represents the mean (+SD) from 3 independent experiments. * *p* < 0.05 (vs. control-siRNA). (**E**) Transwell invasion assay in 4 prostate cancer cell lines transfected with either control-siRNA or ZKSCAN3-siRNA and cultured in the Matrigel-coated transwell chamber for 24 h. The number of invaded cells present in the lower chamber was counted under a light microscope (100× objective in 5 random fields). Cell invasion is presented relative to that of each control line. Each value represents the mean (+SD) from 3 independent experiments. * *p* < 0.05 (vs. control-siRNA). Quantitative real-time RT-PCR of *MMP2* (**F**) or *MMP9* (**G**) in 2 prostate cancer cell lines transfected with either control-siRNA or ZKSCAN3-siRNA. The expression of each specific gene was normalized to that of *GAPDH*, and transcription amount is presented relative to that in each control line. Each value represents the mean (+SD) of 6 determinants. * *p* < 0.05 (vs. control-siRNA).

Using control-siRNA vs. ZKSCAN3-siRNA lines, we assessed the functional role of ZKSCAN3 in the proliferation via methyl-thiazolyl-disphenyl-tetrazolium bromide (MTT) assay (Figure 5A), apoptosis via flow cytometry (Figure 5B) and TUNEL assay (Figure 5C), migration via wound-healing assay (Figure 5D), and invasion via transwell invasion assay (Figure 5E) and quantitative PCR for *MMP2* (Figure 5F) and *MMP9* (Figure 5G) of prostate cancer cells. In these assays, ZKSCAN3 silencing resulted in considerable decreases in cell viability, migration, and invasion, as well as the expression level of *MMP2* or *MMP9*, and considerable increases in apoptosis. In addition, clonogenic assay in C4-2 and PC3 lines where a control-short hairpin RNA (shRNA) or a ZKSCAN3-shRNA was stably expressed (Figure S3A) demonstrated significant decreases in the number and area of colonies in knockdown sublines (Figure S3B).

## 3. Discussion

Although controversial, there have been data showing an association between vasectomy and the subsequent risk of prostate cancer (see Figure S1), especially high-grade and/or lethal tumors [19]. However, because there is no alternative approach for permanent male contraception, it may not be feasible to conduct prospective clinical trials. Meanwhile, these epidemiological findings have not been extensively confirmed by preclinical studies. We therefore investigated the impact of vasectomy on the development and progression of prostate cancer, using animal and cell line models as well as surgical specimens.

An earlier study in C57BL/6 mice showed a higher incidence of spontaneous tumors in the liver, lung, and kidney in vasectomized animals, but none developed prostate cancer [34]. TRAMP mice are known to almost sequentially develop neoplastic lesions in the prostate, such as hyperplasia or PIN (6+ weeks), well differentiated adenocarcinoma (8+ weeks), poorly differentiated adenocarcinoma (16+ weeks), and metastasis (16+ weeks) [31]. As such, we compared the incidence of neoplastic lesions in animals that had undergone vasectomy performed at 4 weeks of age (i.e., prior to the development of PIN) versus those that had not. In all mice sacrificed at 10 weeks, a precursor lesion PIN had developed, and the effect of vasectomy on early tumorigenesis could not be precisely assessed. Nonetheless, poorly differentiated carcinomas occurred significantly more often in vasectomized mice at 24 weeks, compared with controls. Similarly, gross tumor showed a trend towards statistical significance in vasectomized mice at 24 weeks. Moreover, the Ki-67 proliferation index, which has been shown to be a reliable prognosticator for prostate cancer [35,36], was significantly higher in well/moderately carcinomas and poorly differentiated carcinomas from vasectomized TRAMP mice than in those from respective controls. These findings suggest that vasectomy facilitates the development of high-grade/aggressive tumors. However, because only a small subset of TRAMP mice castrated at 12 weeks of age developed poorly differentiated carcinoma (with no metastasis), we were unable to assess whether vasectomy could induce the emergence of aggressive castration-resistant prostate cancer. Meanwhile, in the patient cohort undergoing radical prostatectomy, a history of vasectomy was found to correlate with a significantly higher level of preoperative PSA, but not a significantly higher risk of tumor recurrence. Tumor grades and stages were similar between our two cohorts of prostate cancer patients, those with versus without a known history of vasectomy.

Molecular mechanisms underlying the link between vasectomy and prostate cancer remain speculative. Possible explanations for the increased risk in vasectomy recipients have included: (1) a decrease in the volume of prostatic secretion, resulting in a prolonged exposure to certain carcinogens [37,38]; (2) an increase in circulating androgens or androgen-binding protein binding capacity [4,39]; (3) the development of anti-sperm antibodies or changes in local immune factors all of which may affect immunologic processes [34,40]; and (4) reduced levels of some molecules in the seminal plasma, such as IGF-1 and IGFBP3 that are known to involve prostate carcinogenesis [41]. Upon DNA microarray screening, we revealed that vasectomy considerably induced the expression of ZKSCAN3, a family member of the KRAB and SCAN domain-containing zinc-finger transcription factors, in both non-neoplastic prostate and prostate cancer. Further analyses in radical prostatectomy specimens and prostate cancer cell lines indicated that ZKSCAN3 overexpression was associated with tumor outgrowth. This study thus provides a potential mechanism responsible for the long-term effect of vasectomy on developing prostate cancer and its association with aggressive disease.

ZKSCAN3 has been implicated in the progression of several types of malignancies, including prostate cancer [42], as well as bladder cancer [43], colon cancer [33,44], multiple myeloma [45], and breast cancer [46]. Specifically, in prostate cancer, ZKSCAN3 has been shown to modulate the cell cycle as well as cell attachment, migration, and motility [42]. In other cancer types, the involvement of ZKSCAN3 in modulating cell proliferation/apoptosis and tumorigenicity has also been documented [43,44,46]. The findings in some of these studies [33,43,45,46] have additionally suggested that ZKSCAN3 could modulate several molecules known to play a key role in tumorigenesis and/or tumor progression, such as cyclin D1/D2, EGF, IGF-2, integrin-β4, MMP2/MMP9, NF-κB, and VEGF. Meanwhile, using immunostaining in a commercially available tissue microarray, none of 2 normal prostate tissues versus 49% (38/78) of prostate cancers were found to strongly express ZKSCAN3 [42]. Moreover, *ZKSCAN3* gene amplification was detected in none of 12 primary prostate cancers versus 20% (1/5) of lymph node metastases or 26% (5/19) of bone metastases [42]. Consistent with previous observations, we demonstrated that ZKSCAN3 silencing in prostate cancer lines resulted in significant reduction of cell viability, colony formation, cell migration, cell invasion, and the expression of MMP2/MMP9, as well as significant induction of apoptosis. Our immunohistochemistry in surgical specimens further showed that ZKSCAN3 expression was elevated in prostate cancer, compared with HGPIN, and in HGPIN, compared with non-neoplastic prostate tissue. In addition, ZKSCAN3 expression was found to strongly associate with more aggressive histopathological features and a higher risk of tumor recurrence as an independent predictor.

There are several limitations in our investigation. Specifically, in our surgical prostate resection specimens, available clinical information showed that only 14 of 300 men undergoing radical prostatectomy had a history of vasectomy, and it might thus have been missed in a considerable number of cases. In addition, no data on time factors in vasectomized patients was available, and the relevance of time from vasectomy to cancer development could not be assessed. Similarly, in animal experiments, ZKSCAN3 expression was measured only in 24-week-old C57BL/6 or TRAMP mice that had undergone vasectomy/sham surgery at 10 or 4 weeks of age, respectively, and its chronological changes were not assessed. It therefore remains unanswered why vasectomy has an impact, if any, on prostate cancer development after a > 20-year interval.

## 4. Materials and Methods

### 4.1. Animals

The animal protocol in accordance with National Institutes of Health Guidelines for the Care and Use of Experimental Animals was approved by the Institutional Animal Care and Use Committee (#101492/2012-017). TRAMP and C57BL/6 mice were obtained from The Jackson Laboratory (Bar Harbor, ME, USA). The genotype of TRAMP mice was verified by PCR of tail snip DNA. Either vasectomy or sham surgery was performed in anesthetized male TRAMP mice at the age of 4 weeks. In groups of TRAMP mice, bilateral orchiectomy was additionally performed at 12 weeks. These animals were euthanized at different time points for macroscopic/microscopic analyses (see Figure 1A).

### 4.2. Prostate TMA

We retrieved 300 prostate tissue specimens obtained by radical prostatectomy performed at the University of Rochester Medical Center performed in 2004–2008. Appropriate approval from the Institutional Review Board (#29646), including the request to waive the documentation of informed consent from the patients, was obtained before construction of the TMA consisting of representative lesions of non-neoplastic normal-appearing prostate, HGPIN, and prostatic adenocarcinoma, as we described previously [27,28]. None of the patients had received therapy with hormonal reagents, radiation, or other anticancer drugs pre- or post-operatively before clinical or biochemical recurrence.

### 4.3. Cell Lines

Human prostatic carcinoma cell lines (LNCaP/VCaP/PC3/DU145/C4-2) originally obtained from the American Tissue Type Collection (Manassas, VA, USA) and recently authenticated by the institutional core facility were maintained in RPMI 1640 (Mediatech, Manassas, VA, USA) supplemented with 10% fetal bovine serum (FBS). Gene silencing was achieved by transfection of a ZKSCAN3-siRNA (sc-95093), a control-siRNA (sc-37007), a ZKSCAN3-shRNA (sc-95093-SH), or a control-shRNA (sc-108060) (all from Santa Cruz Biotechnology, Dallas, TX, USA), as we described previously [43,47,48].

### 4.4. Immunohistochemistry

Immunohistochemical staining for Ki-67 (30-9; Ventana) or ZKSCAN3 (TA308508; OriGene, Rockville, MD) was performed in harvested mouse tissues or prostate TMA, respectively, as we described previously [43,49]. All the slides were examined by a single pathologist (H.M.) blinded to sample identify. For Ki-67 staining, its positive rates (as percentages) in cancer cells were determined. For ZKSCAN3 staining, scores (range: 012) were calculated by multiplying the percentage of immunoreactive cells (0% = 0; 110% = 1; 1150% = 2; 5180% = 3; 81100% = 4) by staining intensity (negative = 0; weak = 1; moderate = 2; strong = 3) and were considered negative (0; score < 2), weakly positive (1+; 2 ≤ score ≤ 4), moderately positive (2+; 4 < score ≤ 8), and strongly positive (3+; score > 8).

### 4.5. DNA Microarray

Total RNA extracted from the dorsolateral prostate from two pairs of 24-week-old male C57BL/6 mice undergoing vasectomy or sham surgery at 10 weeks of age, using TRIzol (Invitrogen, Carlsbad, CA, USA), was subjected to microarray gene expression analysis at the University of Rochester Genomics Research Center, using Human Gene 2.0 ST Array (Affymetrix, Santa Clara, CA, USA). Scanned fluorescence signals were converted to continuous values by the Gene Expression Console software (Affymetrix).

### 4.6. Real-Time PCR

Total RNA isolated from prostate tissues or cultured cells was reverse transcribed with Omniscript RT Kit (Qiagen, Germantown, MD, USA) and oligo-dT primers (Qiagen). Real-time PCR was then performed, using SYBR GreenER qPCR SuperMix for iCycler (Invitrogen), as we described previously [43,47,48]. The following primer pairs were used: mouse *ZKSCAN3* (forward, 5′-TGACAGCTACTAGGCTCAC AT-3′; reverse, 5′-GCAAGTCCCTAACCTTAGTCTGC-3′; mouse *GAPDH* (forward, 5′-AATGGATT TGGACGCATTGGT-3′; reverse, 5′-TTTGCACTGGTACGTGTTGAT-3′); human *MMP2* (forward, 5′-TACAGGATCATTGGCTACACACC-3′; reverse, 5′-GGTCACATCGCTCCAGACT-3′); human *MMP9* (forward, 5′-TGTACCGCTATGGTTACACTCG-3′; reverse, 5′-GGCAGGGACAGTTGCTTCT -3′); and human *GAPDH* (forward, 5′-CTCCTCCACCTTTGACGCTG-3′; reverse, 5′-CATACCAGG AAATGAGCTTGACAA-3′).

### 4.7. Western Blotting

Equal amounts of proteins (30-50 µg) obtained from cell extracts were subjected to electrophoresis with 10% sodium dodecyl sulfate-polyacrylamide gel, which was transferred to polyvinylidene difluoride membrane electronically. The membrane was incubated with an anti-ZKSCAN3 antibody (TA308508, dilution 1:500; or A-10, dilution 1:500, Santa Cruz Biotechnology) or an anti-GAPDH antibody (3C5; diluted 1:5000; Santa Cruz Biotechnology), followed by 1 h incubation with a secondary antibody (anti-rabbit IRDye 800CW, LI-COR, Lincoln, NE). Signals were scanned by an infrared imaging system (Odyssey, LI-COR).

### 4.8. Reporter Gene Assay

Cells at a density of 50–70% confluence in 24-well plates were co-transfected with 250 ng of pNFκB-Luc reporter plasmid DNA (LR-2001, Signosis, Santa Clara, CA, USA) and 2.5 ng of a control reporter plasmid (pRL-TK, Addgene, Watertown, MA, USA), using Lipofectamine 300 transfection reagent (Life Technologies, Carlsbad, CA, USA), as we described previously [43,49]. After 24 h of transfection, the cells were harvested, lysed, and assayed for luciferase activity determined using a Dual-Luciferase Reporter Assay kit (Promega, Madison, WI, USA).

### 4.9. Cell Proliferation

We used the MTT assay to assess cell viability. Cells (500–1000/well) seeded in 96-well tissue culture plates were cultured for up to 96 h and then incubated with 0.5 mg/mL of MTT (Sigma-Aldrich, St. Louis, MO, USA) for 3 h at 37 °C. MTT was dissolved by dimethyl sulfoxide, and the absorbance at 570 nm was measured.

### 4.10. Clonogenic Assay

Cells (500/well) seeded in 12-well tissue culture plates were allowed to grow until colonies in the control well were easily distinguishable. The cells were fixed with methanol and stained with 0.1% crystal violet. The number and area of colonies in photographed pictures were then quantitated, using ImageJ software (National Institutes of Health, Bethesda, MD, USA).

### 4.11. Apoptosis

Apoptosis was assessed by two methods. First, flow cytometry was performed in cells (1 × 10^6^/10-cm dish) cultured for 24 h, harvested, fixed in 70% ethanol, and stained with PI buffer (50 μg/mL). Cellular PI content was measured on a Guava PCA-96 Base System flow cytometer (EMD Millipore, Burlington, MA, USA) equipped with a green laser at 532 nm wave length. Second, the TUNEL assay was conducted on cell-burdening coverslips, using DeadEnd Fluorometric TUNEL system (Promega), followed by counterstaining for DNA with 4′,6-diamidino-2-phenylindole. Apoptotic index was determined in the cells visualized by the fluorescence microscopy.

### 4.12. Cell Migration

Scratch wound-healing assay was adapted to evaluate the ability of cell migration. Cells at a density of ≥90% confluence in 12-well tissue culture plates were scratched manually with a sterile 200 μL plastic pipette tip. The wounded monolayers of the cells were allowed to heal in serum-free medium for 24 h. The normalized cell-free area was then quantitated, using the ImageJ.

### 4.13. Cell Invasion

Cell invasiveness was determined, using a Matrigel-coated transwell chamber (8.0-μm pore size polycarbonate filter with 6.5-mm in diameter; Corning Inc., Corning, NY, USA). Cells (5 × 10^3^) in 100 μL of serum-free medium were added to the upper chamber of the transwell, whereas 600 μL of medium containing 5% FBS was added to the lower chamber. After incubation for 24 h, invaded cells were fixed, stained with 0.1% crystal violet, and counted.

### 4.14. Statistical Analysis

Fisher’s exact test or chi-square test was used to evaluate the associations between categorized variables. The numerical data were compared by Student’s *t*-test. Survival rates in patients were calculated by the Kaplan-Meier method, and comparison was made by the log-rank test. The Cox proportional hazards model was used to determine statistical significance in a multivariate setting. *p* < 0.05 was considered to be statistically significant.

## 5. Conclusions

The present findings in a transgenic mouse model indicated that vasectomy might induce the development of prostate cancer in certain circumstances, specifically aggressive tumor. Vasectomy was also found to enhance the expression of a transcription factor ZKSCAN3 in the prostate. Furthermore, ZKSCAN3 was suggested to play an important role in the development and progression of prostate cancer. These results not only support epidemiological data indicating an association between vasectomy and subsequent prostate cancer risk but may also offer a potential chemopreventive or therapeutic strategy for prostate cancer, via targeting ZKSCAN3 signaling, especially in vasectomized men.

## Figures and Tables

**Table 1:** Incidence of tumors in TRAMP mice.

	10 Weeks			16 Weeks			24 Weeks		
	Sham (*n* = 15)	Vasectomy (*n* = 15)	*p*	Sham (*n* = 12)	Vasectomy (*n* = 12)	*p*	Sham (*n* = 12)	Vasectomy (*n* = 12)	*p*
**Gross tumor**	0 (0%)	0 (0%)	1.000	0 (0%)	2 (17%)	0.478	2 (17%)	7 (58%)	0.089
**Carcinoma**	9 (60%)	7 (47%)	0.715	8 (67%)	7 (58%)	1.000	12 (100%)	12 (100%)	1.000
Well/moderately differentiated	9 (60%)	7 (47%)	0.715	8 (67%)	7 (58%)	1.000	10 (83%)	4 (33%)	0.036
Poorly differentiated	0 (0%)	0 (0%)	1.000	0 (0%)	0 (0%)	1.000	2 (17%)	8 (67%)	0.036
**Metastasis**	0 (0%)	0 (0%)	1.000	0 (0%)	0 (0%)	1.000	2 (17%)	4 (33%)	0.640

**Table 2:** Expression levels of ZKSCAN3 in radical prostatectomy specimens.

	*n*	Levels of ZKSCAN3 Expression					*p*			IHC Score (mean ± SD)	*p* (*t*-Test)
		0	1+	2+	3+	1+/2+/3+	0 vs. 1+/2+/3+	0/1+ vs. 2+/3+	0/1+/2+ vs. 3+		
Benign	150	99 (66%)	44 (29%)	6 (4%)	1 (1%)	51 (34%)	<0.001 *^1^	<0.001 *^1^	0.301 *^1^	1.65 ± 1.41	<0.001 *^1^
HGPIN	146	54 (37%)	68 (47%)	21 (14%)	3 (2%)	92 (63%)	<0.001 *^2^	<0.001 *^2^	<0.001 *^2^	2.58 ± 1.66	<0.001 *^2^
Carcinoma	300	21 (7%)	99 (33%)	95 (32%)	85 (28%)	279 (93%)	<0.001 *^3^	<0.001 *^3^	<0.001 *^3^	5.20 ± 2.33	<0.001 *^3^
Age (mean ± SD, year)	300	58.0 ± 4.8	60.2 ± 6.4	60.2 ± 6.1	60.8 ± 6.1	60.4 ± 6.2	0.079	0.363	0.275	NA	
PSA (mean ± SD, ng/mL)	300	5.72 ± 3.27	6.02 ± 3.07	7.21 ± 4.88	6.44 ± 3.87	6.31 ± 3.91	0.441	0.243	0.623	NA	
Gleason score (GS)/Grade Group (GG)											
GS ≤ 6/GG 1	107	11 (10%)	32 (30%)	44 (41%)	20 (19%)	96 (90%)	0.097 *^4^	0.961 *^4^	0.006 *^4^	4.91 ± 2.15	0.099 *^4^
GS 3 + 4 = 7/GG 2	127	9 (7%)	52 (41%)	36 (28%)	30 (24%)	118 (93%)	0.048 *^5^	0.003 *^5^	<0.001 *^5^	4.85 ± 2.34	<0.001 *^5^
GS 4 + 3 = 7/GG 3	46	1 (2%)	11 (24%)	9 (20%)	25 (54%)	45 (98%)	0.204 *^6^	0.059 *^6^	0.026 *^6^	6.26 ± 2.28	0.010 *^6^
GS ≥ 8/GG 4–5	20	0 (0%)	4 (20%)	6 (30%)	10 (50%)	20 (100%)				6.50 ± 2.09	
Pathologic stage (pT)											
2	235	15 (6%)	88 (37%)	79 (34%)	53 (23%)	220 (94%)	0.426 *^7^	0.010 *^7^	<0.001 *^7^	4.95 ± 2.19	<0.001 *^7^
3a	47	6 (13%)	9 (19%)	13 (28%)	19 (40%)	41 (87%)	0.230 *^8^	0.010 *^8^	<0.001 *^8^	5.51 ± 2.58	<0.001 *^8^
3b	18	0 (0%)	2 (11%)	3 (17%)	13 (72%)	18 (100%)				7.61 ± 1.90	
Lymph node metastasis (pN)							0.326	0.075	0.021		0.002
0	154	15 (10%)	48 (31%)	45 (29%)	46 (30%)	139 (90%)				5.05 ± 2.46	
1	9	0 (0%)	1 (11%)	2 (22%)	6 (67%)	9 (100%)				7.67 ± 1.94	
History of vasectomy							0.293	0.044	0.217		0.018
No	286	21 (7%)	97 (34%)	89 (31%)	79 (28%)	265 (93%)				5.13 ± 2.33	
Yes	14	0 (0%)	2 (14%)	6 (43%)	6 (43%)	14 (100%)				6.64 ± 1.79	

*^1^ Benign vs. HGPIN. *^2^ HGPIN vs. Carcinoma. *^3^ Benign vs. Carcinoma. *^4^ GG1 vs. GG2-5. *^5^ GG1-2 vs. GG3-5. *^6^ GG1-3 vs. GG4-5. *^7^ pT2 vs. pT3. *^8^ pT2/pT3a vs. pT3b.

## Supplementary Materials

The following are available online at https://www.mdpi.com/2072-6694/12/8/2295/s1, Figure S1: The status of vasectomy and relative risks for prostate cancer in US cohort/case-control studies., Figure S2: The whole Western blotting images for Figure 4A,B, Figure S3: Effect of ZKSCAN3 knockdown on colony formation, Table S1: Incidence of tumors in castrated TRAMP mice., Table S2: Vasectomy and radical prostatectomy findings.
