# Supplementary material for: Impact of Vasectomy on the Development and Progression of Prostate Cancer: Preclinical Evidence
Source: Cancers (Basel). 2020 Aug 15;12(8):2295. doi: 10.3390/cancers12082295 (PMC7464827; doi:10.3390/cancers12082295)
Supplement: Supplementary file 1 [file cancers-12-02295-s001.pdf]

Supplementary Materials

Table S1. Incidence of tumors in castrated TRAMP mice.

	**Sham** (***n*** **= 13)**	**Vasectomy** (***n*** **= 13)**	***p*-Value**
**Gross tumor**	0 (0%)	1 (8%)	1.000
**Carcinoma**	3 (23%)	4 (31%)	1.000
Well/moderately differentiated	1 (8%)	3 (23%)	0.593
Poorly differentiated	2 (15%)	1 (8%)	1.000
**Metastasis**	0 (0%)	0 (0%)	1.000

Table S2. Vasectomy and radical prostatectomy findings.

	**Vasectomy** (***n*** **= 14)**	**No vasectomy** (***n*** **= 286)**	***p*-Value**
**Age** (mean ± SD, yr)	60.1 ± 4.6	60.2 ± 6.2	0.926
**PSA** (mean ± SD, ng/mL)	8.95 ± 8.76	6.14 ± 3.44	0.008
**Gleason score (GS) / Grade Group (GG)**			
GS ≤ 6 / GG 1	3 (21%)	104 (36%)	0.255*1
GS 3 + 4 = 7 / GG 2	8 (57%)	119 (42%)	0.958*2
GS 4 + 3 = 7 / GG 3	2 (14%)	44 (15%)	0.942*3
GS ≥ 8 / GG 4-5	1 (7%)	19 (7%)	
**Pathologic stage (pT)**			
2	10 (71%)	225 (79%)	0.521*4
3a	2 (14%)	45 (16%)	0.181*5
3b	2 (14%)	16 (6%)	
**Lymph node metastasis (pN)**			0.299*6
0	6 (43%)	148 (52%)	
1	1 (7%)	8 (3%)	
X	7 (50%)	130 (45%)	

*1GG1 vs. GG2-5. *2GG1-2 vs. GG3-5. *3GG1-3 vs. GG4-5. *4pT2 vs. pT3. *5pT2/pT3a vs. pT3b. *6pN0 vs. pN1.

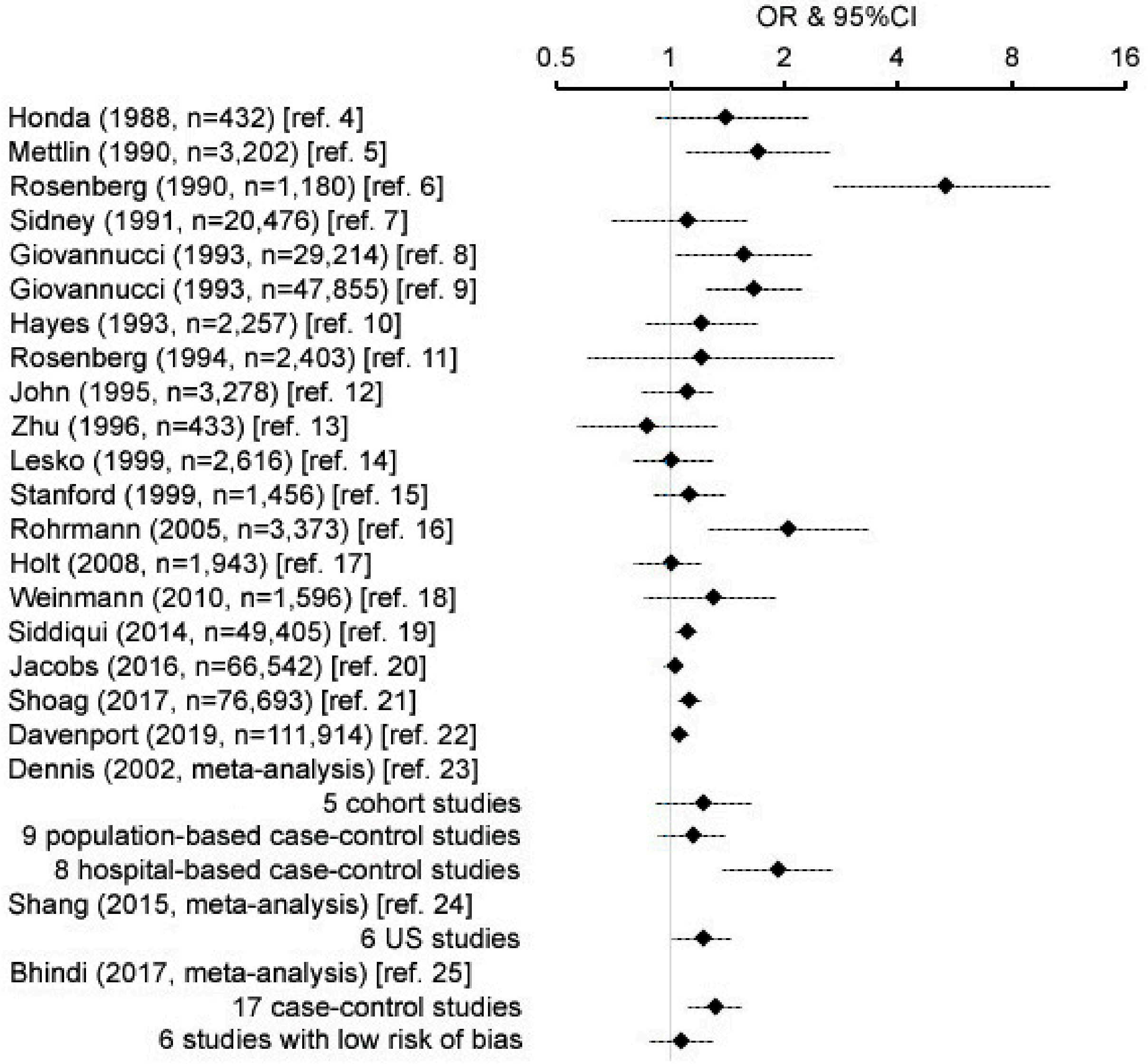

Figure S1. The status of vasectomy and relative risks for prostate cancer in US cohort/case-control studies.

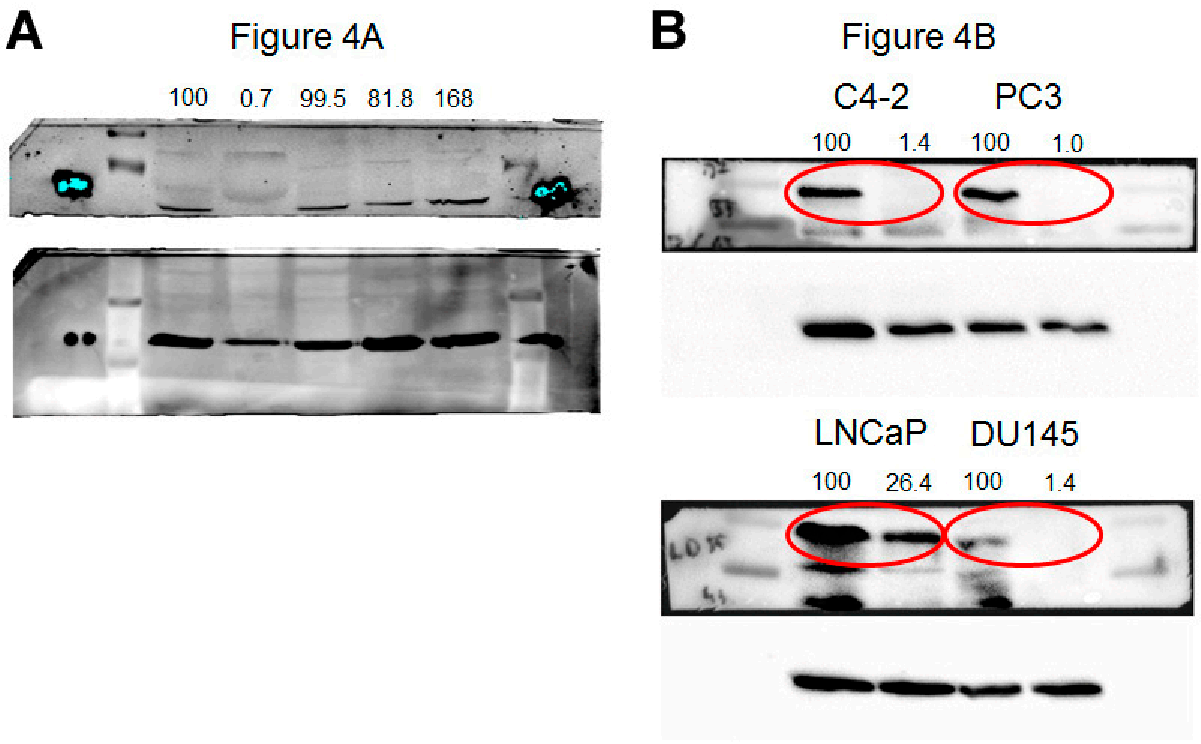

Figure S2. The whole Western blotting images for Figures 4A (**A**) and 4B (**B**). In all of these Western blots, the PVDF membrane was cut before incubation with primary antibodies, including those against ZKSCAN3 and GAPDH. Densitometry values standardized by GAPDH [that are relative to those of LNCaP (Figure 4A) or each control-siRNA subline (Figure 4B)] are shown.

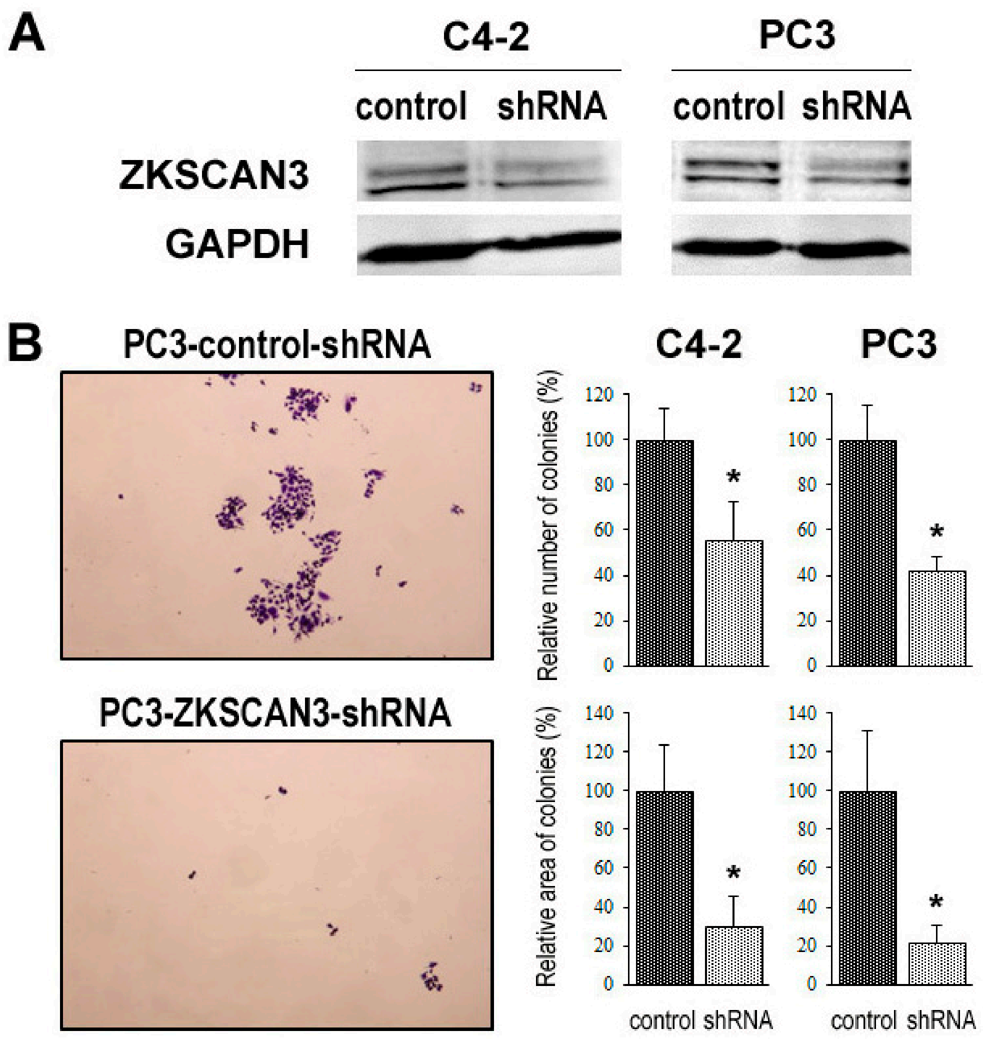

Figure S3. Effects of ZKSCAN3 knockdown on colony formation. (**A**) Western blotting of ZKSCAN3 (60 kDa) in 2 prostate cancer lines stably expressing either control-shRNA or ZKSCAN3-shRNA. GAPDH (37 kDa) served as an internal control. (**B**) Clonogenic assay in C4-2-control-shRNA vs. C4-2-ZKSCAN3-shRNA and PC3-control-shRNA vs. PC3-ZKSCAN3-shRNA. The number of colonies and their areas quantitated, using the ImageJ software, are presented relative to those of each control line. Each value represents the mean (+SD) of 6 determinants. * $p < 0.05$ (vs. control-shRNA).
